# Supplementary material for: Engineered immunogens to elicit antibodies against conserved coronavirus epitopes
Source: Nat Commun. 2023 Nov 30;14:7897. doi: 10.1038/s41467-023-43638-9 (PMC10689493; doi:10.1038/s41467-023-43638-9)
Supplement: Supplementary file 1 — Supplementary Information [file 41467_2023_43638_MOESM1_ESM.pdf]

Supplementary Online Materials

Supplementary Table 1: Data collection and refinement statistics

	S2hlx_EX_19	S2hlx_7-DH1057.1	FP15_DH1058
**Data collection**			
Space group	*C*2	*P*1	$P2_1$
Cell dimensions			
a, *b*, *c* (Å)	175.62, 28.66, 65.49	56.68, 76.36, 79.02	48.39, 50.58, 146.24
α, β, γ (°)	90.00, 106.66, 90.00	71.60, 69.99, 88.30	90.00, 96.89, 90.00
Resolution (Å)	44.55-2.30 (2.38-2.30) *	45.68-1.90 (1.97-1.90)	47.33-2.20 (2.28-2.20)
R_{merge}	0.705 (1.651)	0.664 (0.933)	0.745 (1.051)
$I / \sigma I$	16.1 (2.4)	29.1 (3.2)	23.6 (2.3)
Completeness (%)	95.1 (92.6)	93.9 (87.6)	98.2 (98.6)
Redundancy	13.5 (14.0)	4.7 (3.7)	7.5 (7.5)
**Refinement**			
Resolution (Å)	44.55-2.30 (2.38-2.30)	44.66-1.90 (1.96-1.90)	47.33-2.20 (2.27-2.20)
No. reflections	13,713 (1,340)	87,255 (8,122)	35,397 (3,494)
R_{work} / R_{free}	0.254/0.275	0.211/0.245	0.189/0.229
No. atoms			
Protein	2,212	8,003	4,264
Ligand/ion	0	111	0
Water	69	1,353	189
B-factors			
Protein	45.6	24.7	61.6
Ligand/ion		44.0	
Water	46.4	31.6	49.5
R.m.s. deviations			
Bond lengths (Å)	0.007	0.008	0.006
Bond angles (°)	1.00	0.86	0.85

*Values in parentheses are for highest-resolution shell.

	Patient #	Vaccine	Days post vaccination	COVID strain	Variant of concern	Days post symptom onset
Vaccinated	1	Ad26.Cov2.S plus BNT162b2	42			
	2	mRNA-1273	28			
	3	mRNA-1273	29			
	4	mRNA-1273	20			
	5	mRNA-1273	14			
	6	mRNA-1273	44			
	7	mRNA-1273	13			
	8	BNT162b2	52			
	9	BNT162b2	33			
	10	BNT162b2	26			
	11	BNT162b2	26			
	12	BNT162b2	33			
Infected	13			B.1.2		21
	14			B.1.2		28
	15			AY.103	DELTA	14
	16			AY.118	DELTA	21
	17			AY.44	DELTA	21
	18			AY.118	DELTA	28
	19			AY.44	DELTA	28
	20			BA.1.1	OMICRON	21
	21			BA.1.1	OMICRON	21
	22			BA.1.1	OMICRON	28
	23			BA.1.1	OMICRON	28
	24			BA.1.1	OMICRON	7
Vaccinated + Infected	25	Ad26.Cov2.S	51	AY.103	DELTA	14
	26	Ad26.Cov2.S	192	AY.44	DELTA	21
	27	Ad26.Cov2.S	137	AY.103	DELTA	21
	28	Ad26.Cov2.S	96	AY.44	DELTA	28
	29	Ad26.Cov2.S	193	BA.1.1	OMICRON	28
	30	mRNA-1273	376	BA.1.1	OMICRON	21
	31	BNT162b2	65	B.1.1.207		28
	32	BNT162b2	38	AY.44	DELTA	21
	33	BNT162b2	131	AY.44	DELTA	28
	34	BNT162b2	41	AY.44	DELTA	28
	35	BNT162b2	516	BA.2.12.1	OMICRON	28
	36	BNT162b2	437	BA.2.12.1	OMICRON	28

Supplementary Table 2. SARS-CoV-2 strain and vaccine information of patient samples analyzed for binding to native protein domains and the engineered epitope scaffolds.

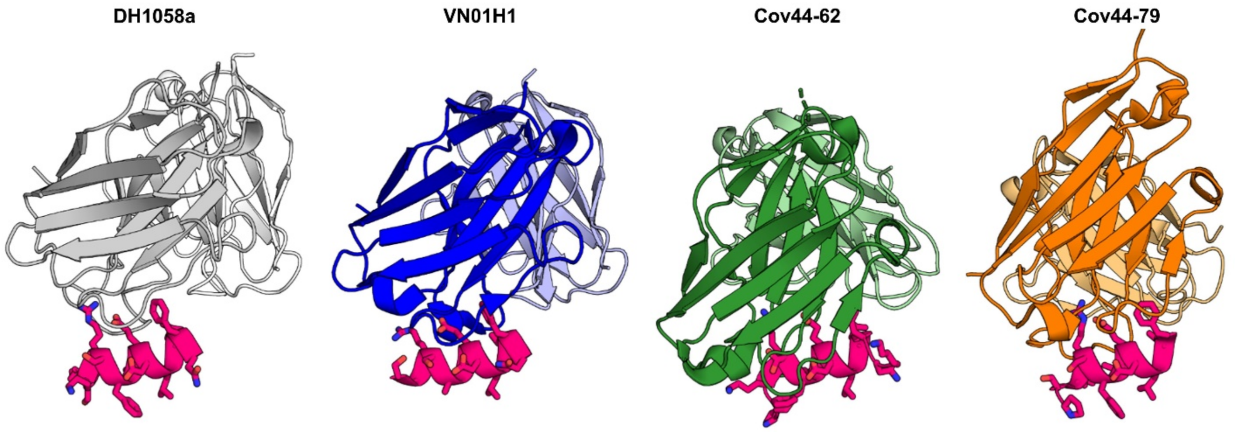

Supplementary Figure 1. Antibodies with broad recognition against diverse coronaviruses target the conserved spike$^{815\text{-}823}$ epitope of SARS-CoV-2. Known structures of DH1058 (*grey*), VN01H1 (*blue*), Cov44-62 (*green*), and Cov44-79 (*orange*) mAbs bound to their spike epitope (*magenta*). Epitopes are centered on residues 813-824 with key contacts made with virus residues R815, E819 and F823.

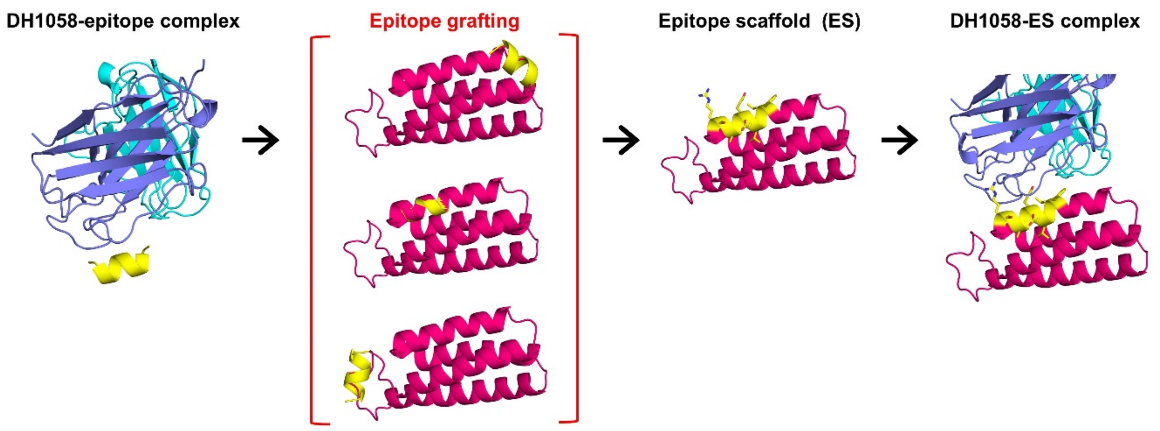

Supplementary Figure 2. Workflow for computational design of spike$^{815-823}$ epitope scaffolds by side chain grafting. Using the crystal structure of antibody DH1058 (*light purple* and *cyan*) bound to its epitope (*yellow*), candidate scaffolds (*magenta*) were queried computationally to identify proteins with exposed backbone regions that closely matched (<0.5Å RMS) the structure of the antibody-bound epitope. On proteins that have regions with high structural mimicry to the DH1058-bound epitope, the epitope sequence (*yellow*) replaced the native scaffold to generate an epitope scaffold (ES); additional mutations were introduced into the scaffold to accommodate the grafted epitope, to prevent clashes with the DH1058 mAb in the modeled antibody-ES complex, and to revert any unnecessary scaffold mutations introduced at the automated computational design stage.

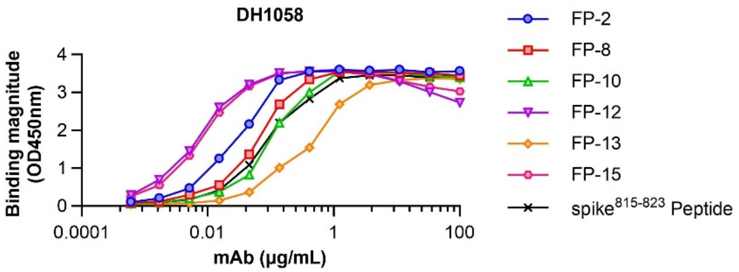

Supplementary Figure 3. Initial binding screen of designed spike$^{815-823}$ epitope scaffolds to DH1058 mAb. ELISA binding of DH1058 mAb to immobilized epitope scaffolds and a synthetic peptide encompassing the spike$^{815-823}$ epitope (SARS-CoV-2 spike residues 808-833). Source data are provided as a Source Data file.

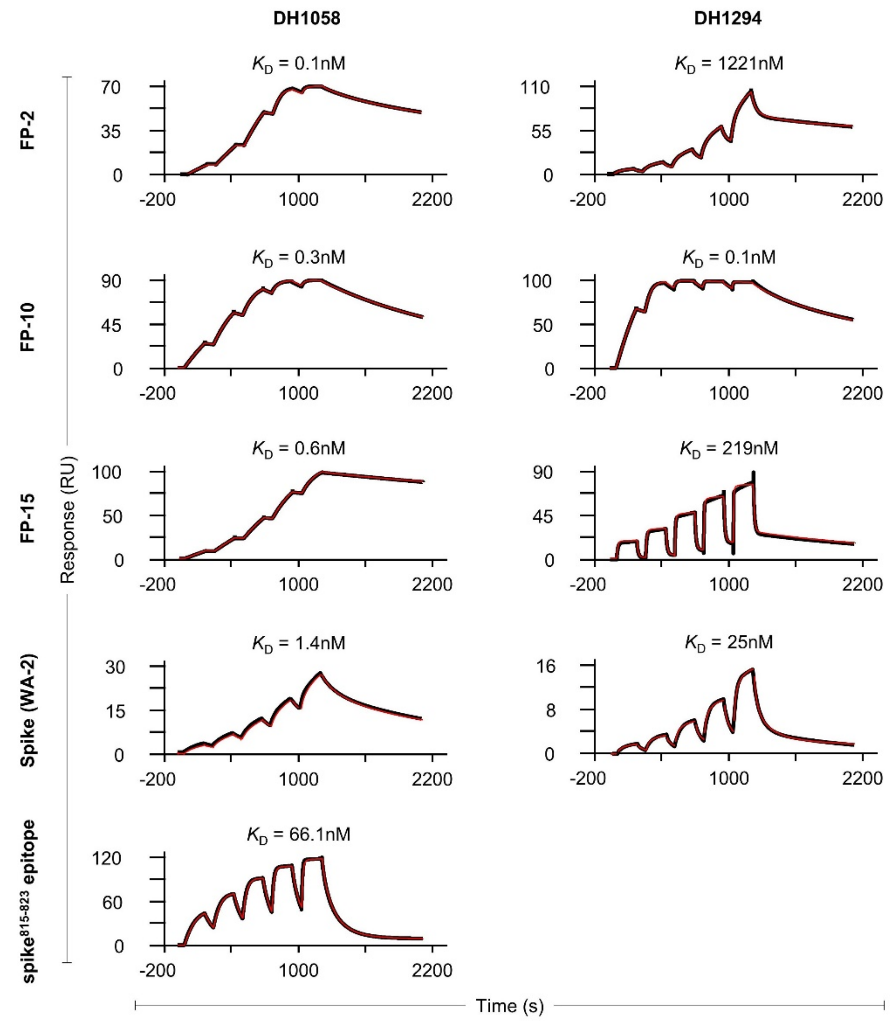

Supplementary Figure 4. Surface Plasmon Resonance binding curves to determine the dissociation constants of spike$^{815\text{-}823}$ epitope scaffolds for DH1058 and DH1294 mAbs. Acquired data is shown in *black* and the curve fit is in *red*. Data is representative of at least two independent experiments.

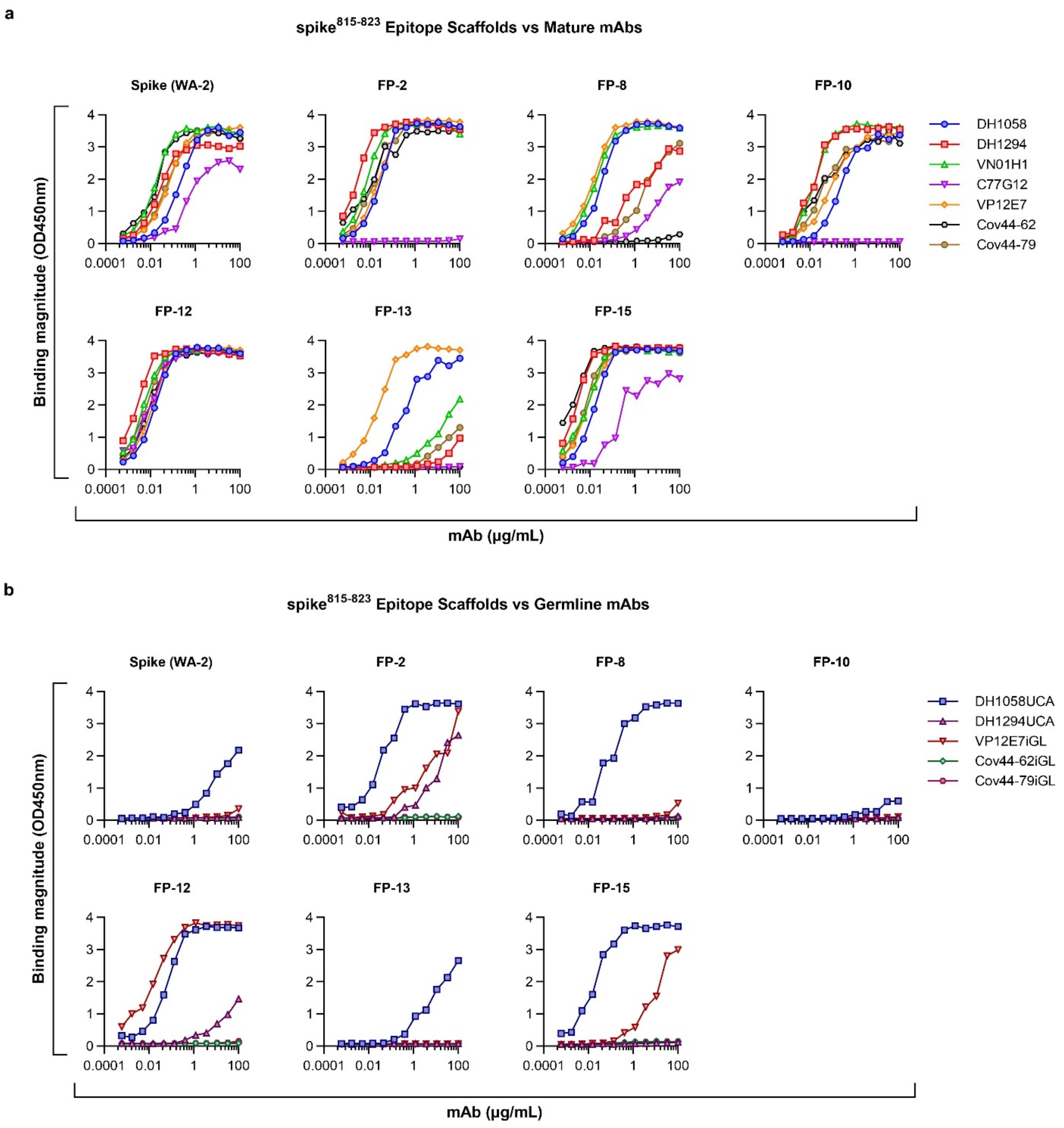

Supplementary Figure 5. Epitope scaffolds bind to diverse mature and germline antibodies that target the spike$^{815-823}$ epitope. (a) ELISA binding of mAbs DH1058, DH1294, VN01H1, C77G12, VP12E7, Cov44-62, and Cov44-79 to FP ESs and to a synthetic peptide encompassing the spike$^{815-823}$ epitope (SARS-CoV-2 spike residues 808-833). **(b)** ELISA binding of mAbs DH1058UCA, DH1294UCA, VP12E7iGL, Cov44-62iGL, and Cov44-79iGL to FP ESs

and to a synthetic peptide encompassing the spike$^{815-823}$ epitope (SARS-CoV-2 spike residues 808-833). Source data are provided as a Source Data file.

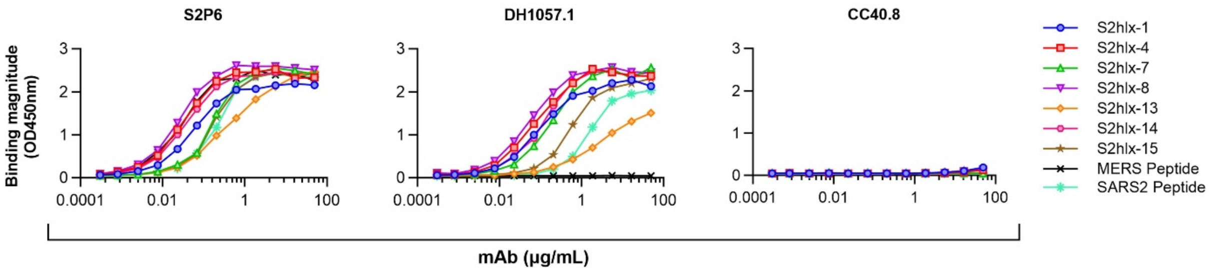

Supplementary Figure 6. S2hlx ESs bind to S2P6 and DH1057.1 mAbs, but not to CC40.8 mAb. ELISA binding of mAbs S2P6, DH1057.1 and CC40.8 to S2hlx ESs measured by ELISA. Binding was compared to that of synthetics stem helix peptides derived from SARS-CoV2 (^{1147}SFKEELDKYFKNHTS^{1161}) and MERS (^{1230}DFQDELDEFFKNVST^{1244}). The synthetic peptide did not contain residue L1145 (SARS-CoV-2 numbering) that is critical for epitope binding by CC40.8. Source data are provided as a Source Data file.

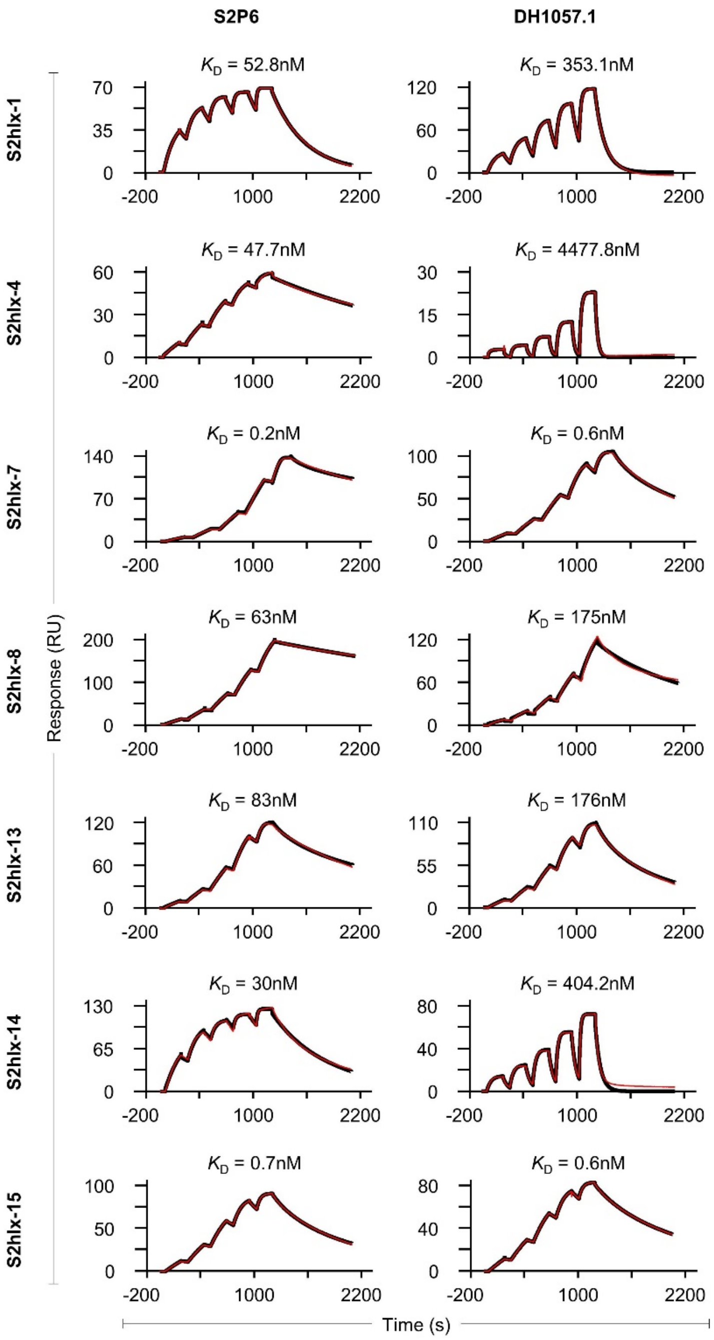

Supplementary Figure 7. Surface Plasmon Resonance curves of S2hlx epitope scaffolds binding to S2P6, S2P6iGL, DH1057.1 and DH1057UCA mAbs. Acquired data is shown in *black* and the curve fit is in *red*.

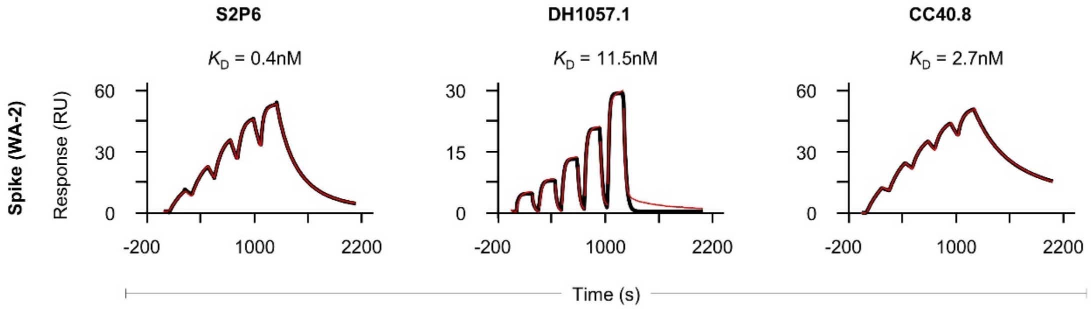

Supplementary Figure 8. Surface Plasmon Resonance curves of Spike (WA-2) proteins binding to S2P6, DH1057.1 and CC40.8 mAbs. Acquired data is shown in *black* and the curve fit is in *red*.

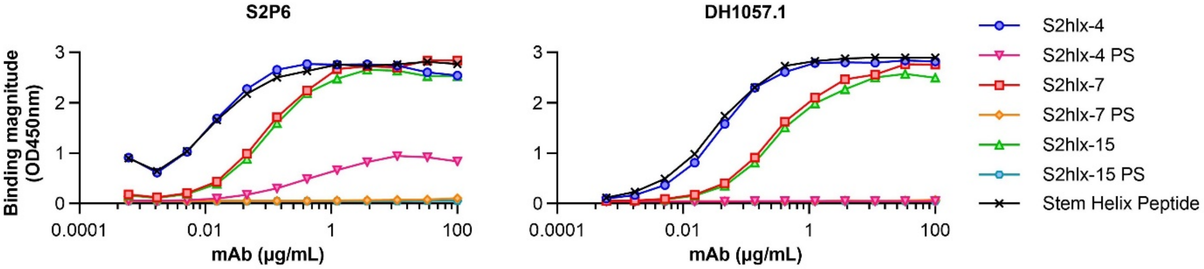

Supplementary Figure 9. Binding of S2hlx epitope scaffolds to target antibodies is mediated by the grafted epitope residues. ELISA binding of S2P6 and DH1057.1 mAbs to S2hlx epitope scaffolds and to the parent scaffolds (PS) that served as templates for epitope grafting. Source data are provided as a Source Data file.

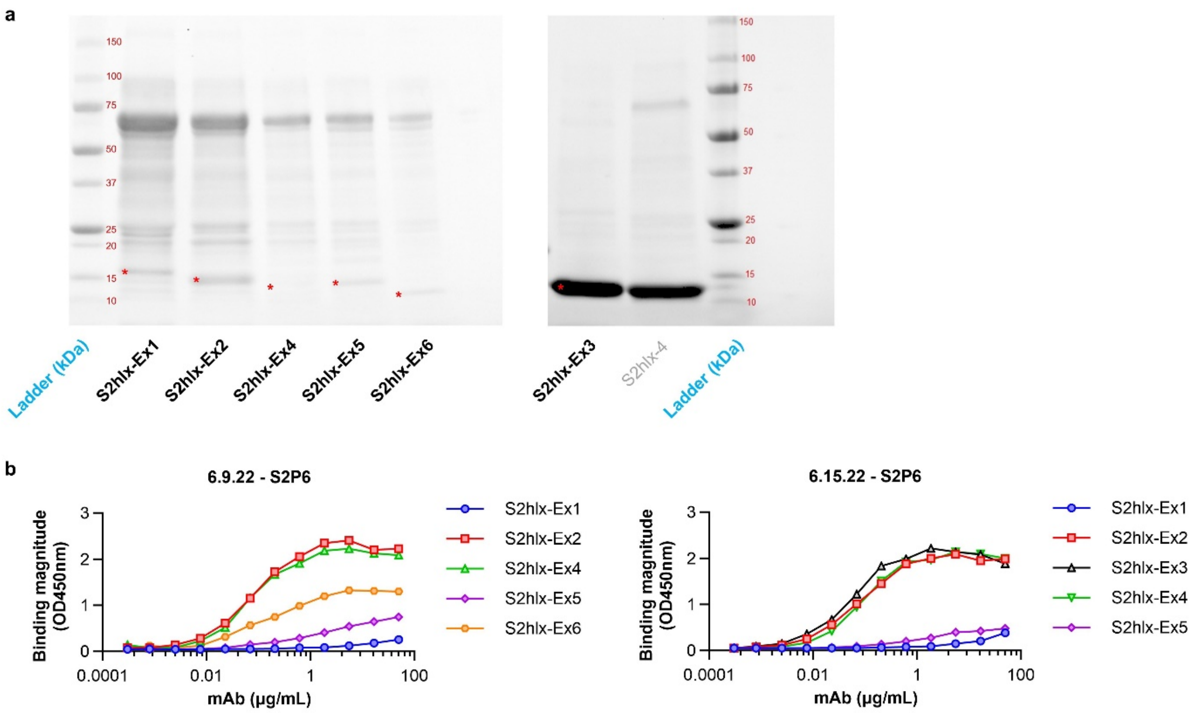

Supplementary Figure 10. Recombinant expression and S2P6 mAb binding of S2hlx-Ex epitope scaffolds designs generated by backbone grafting. (a) SDS-PAGE gel showed limited expression of initial S2hlx-Ex epitope scaffolds. *marks correct MW position for scaffold. Images are representative of at least 2 independent replicates. **(b)** Binding of mAb S2P6 to immobilized S2hlx-Ex epitope scaffolds measured by ELISA. Source data are provided as a Source Data file.

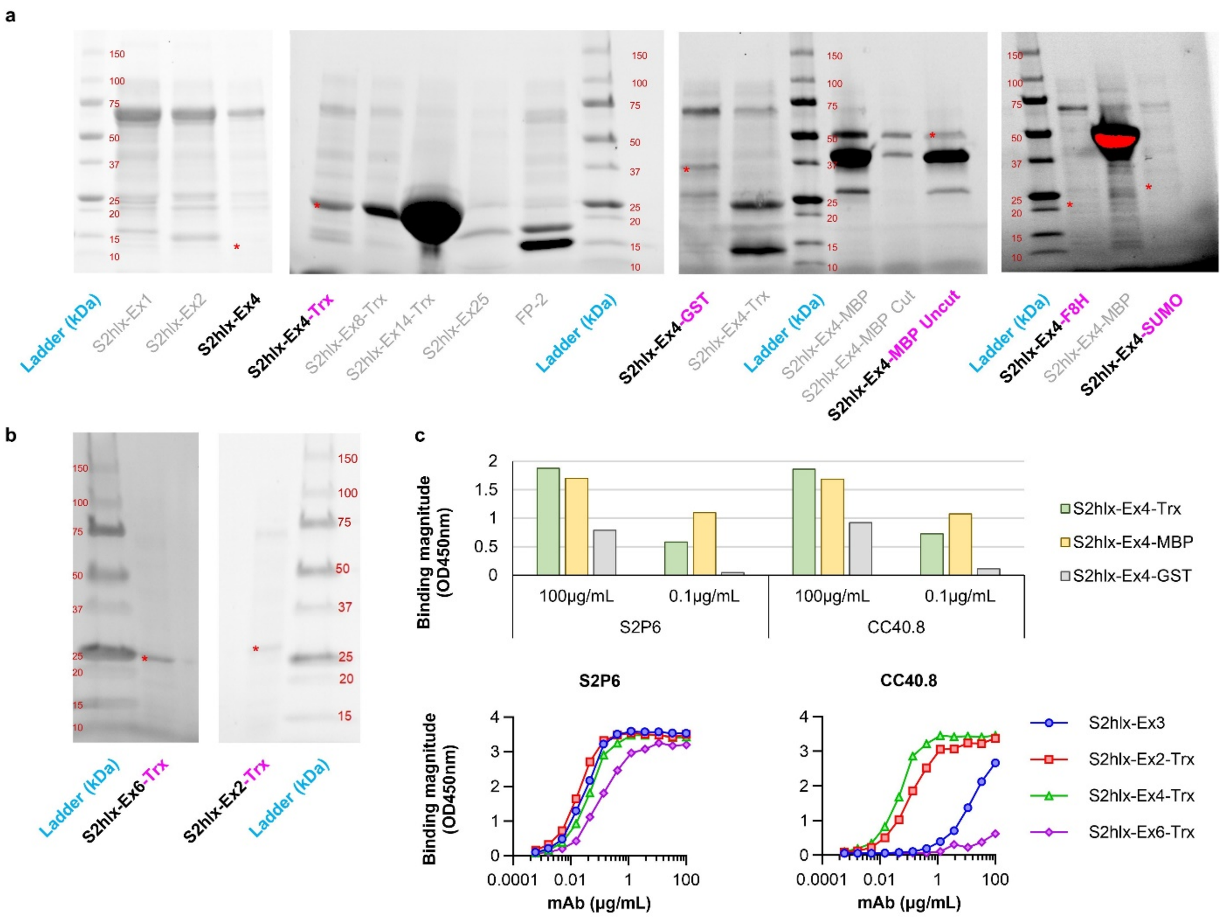

Supplementary Figure 11. Tagged S2hlx-Ex epitope scaffolds showed improved expression and antibody binding. (a) SDS-PAGE gel showing the expression of the initial S2hlx-Ex4 design and of different versions fused to the tags highlighted in *magenta*. Images are representative of at least 2 independent replicates. **(b)** SDS-PAGE gel showing expression of Trx-tagged versions of S2hlx-Ex2, Ex4, and Ex6. * marks expected molecular weight bands. **(c)** Binding of S2P6 and CC40.8 mAbs to tagged S2hlx-Ex epitope scaffolds measured by ELISA. Source data are provided as a Source Data file.

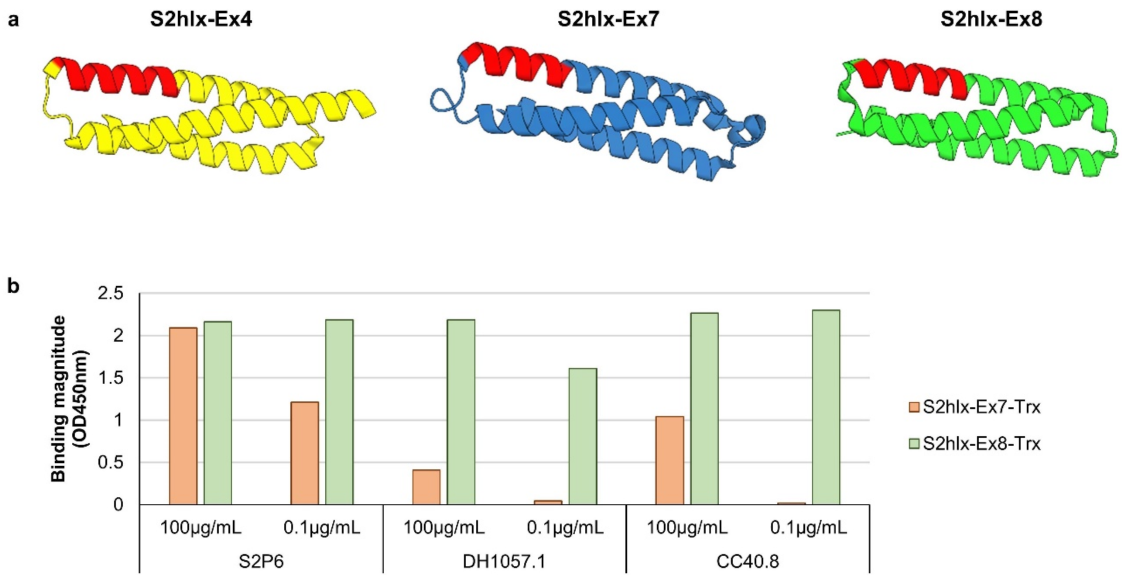

Supplementary Figure 12. Binding of epitope scaffolds designed on structural homologs of the S2hlx-Ex4 parent scaffold. (a) Structures of the S2hlx-Ex4 epitope scaffold and of two related designs, S2hlx-Ex7 and S2hlx-Ex8, based on structurally homologous parent scaffolds (PDBids: 2qyw, 1lvf, and 3onj). The grafted epitope is shown in *red*. **(b)** ELISA binding of antibodies S2P6, DH1057, and CC40.8 to tagged epitope scaffolds from **(a)**. Source data are provided as a Source Data file.

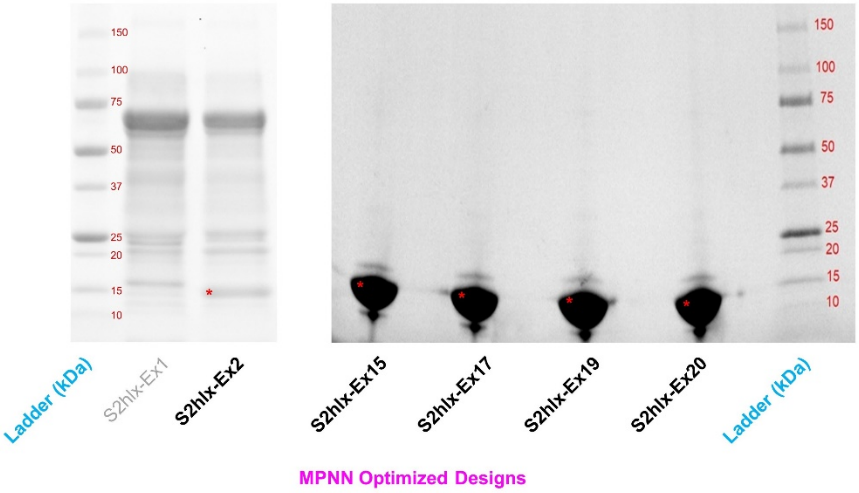

Supplementary Figure 13. Expression of S2hlx-Ex2 and related successful MPNN designs. SDS-PAGE gel showing the expression of the initial S2hlx-Ex2 design and of different MPNN versions. *marks correct MW position for scaffold. Images are representative of at least 2 independent replicates. Source data are provided as a Source Data file.

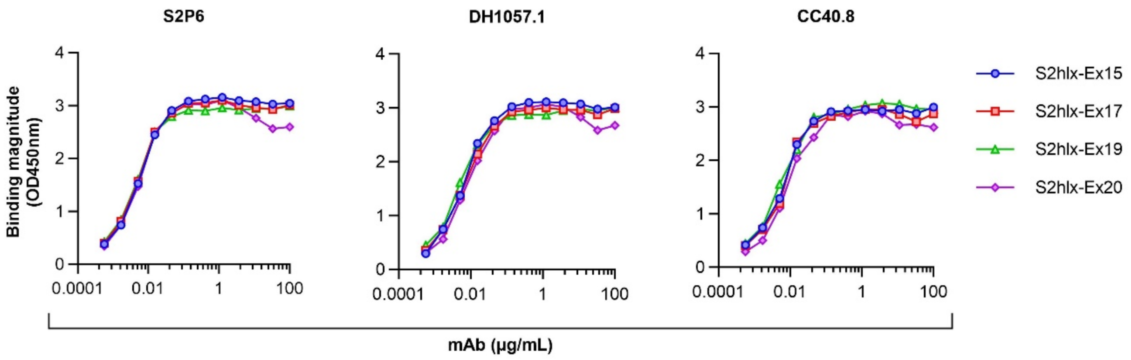

Supplementary Figure 14. S2hlx-Ex2 based MPNN designs show broad binding. Binding of S2P6, DH1057.1 and CC40.8 mAbs to MPNN designs based on S2hlx-Ex2 epitope scaffolds measured by ELISA. Source data are provided as a Source Data file**.**

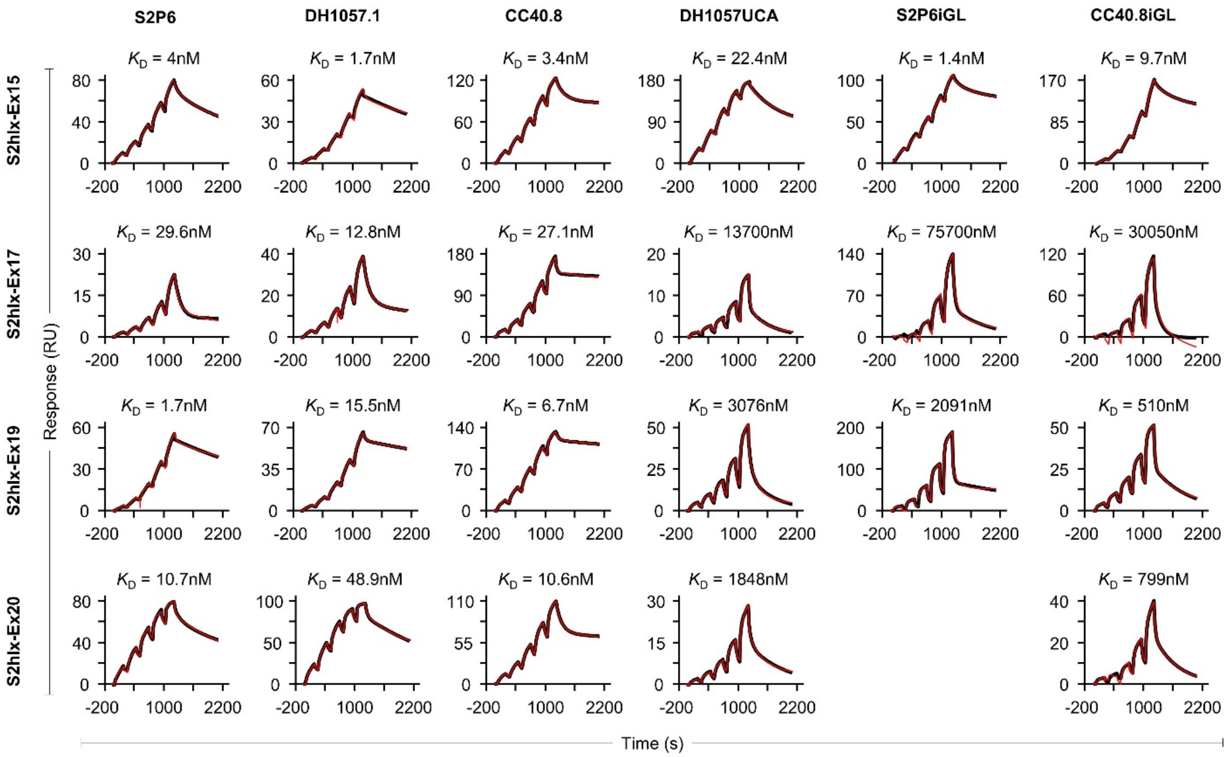

Supplementary Figure 15. Surface Plasmon Resonance curves of S2hIx-Ex2 derived epitope scaffolds binding to S2P6, S2P6iGL, DH1057.1, DH1057UCA, CC40.8 and CC40.8iGL antibodies. Binding kinetics of S2hIx-Ex2 derived epitope scaffolds to S2P6, DH1057, and CC40.8 mAbs and to their germline variants as measured by Surface Plasmon Resonance (SPR).

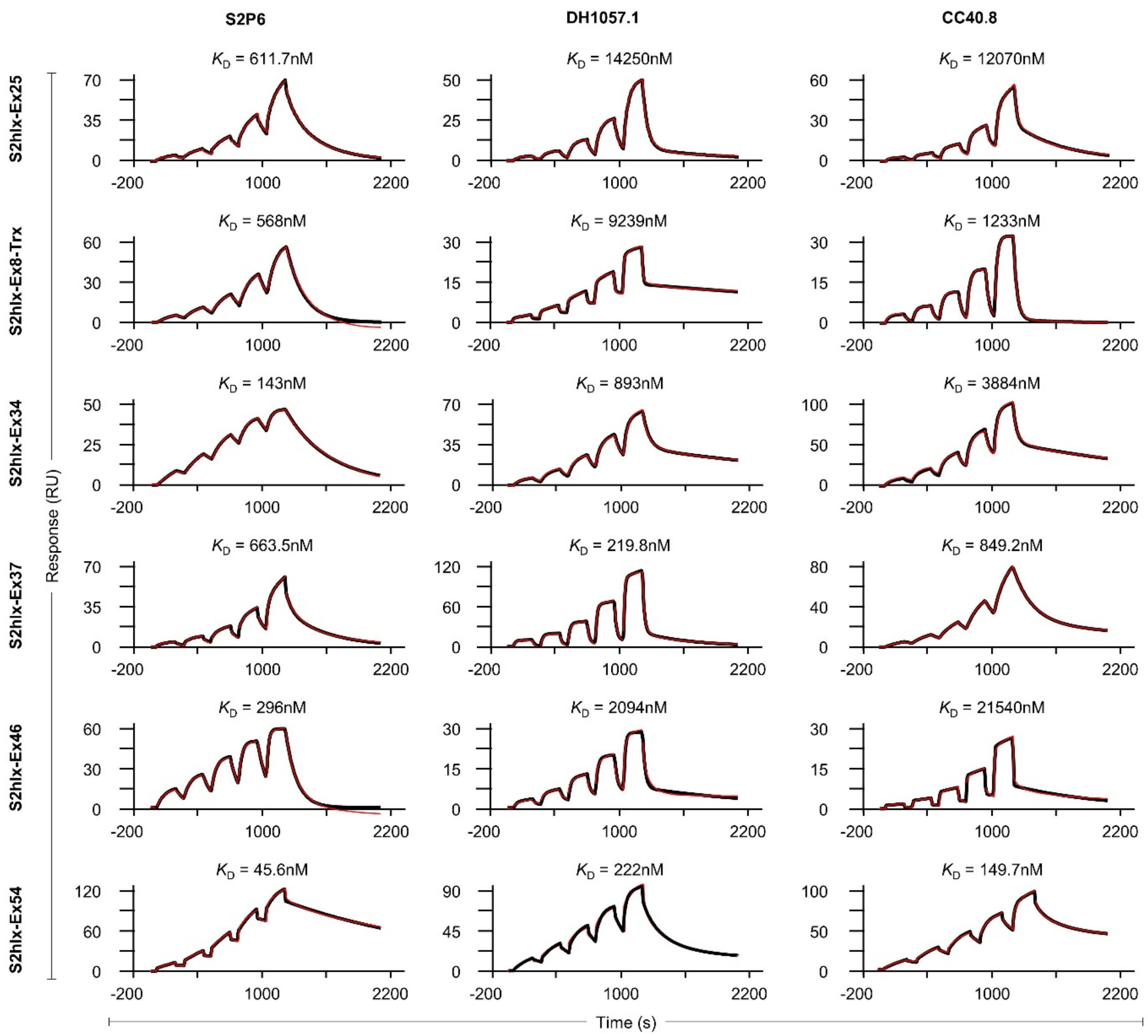

Supplementary Figure 16. Binding affinities of S2hlx-Ex4, Ex6, and Ex3 derived epitope scaffolds to S2P6, DH1057.1, and CC40.8 mAbs. Binding kinetics of S2hlx-Ex4, Ex6, and Ex3 derived epitope scaffolds to S2P6, DH1057.1, and CC40.8 as measured by Surface Plasmon Resonance (SPR).

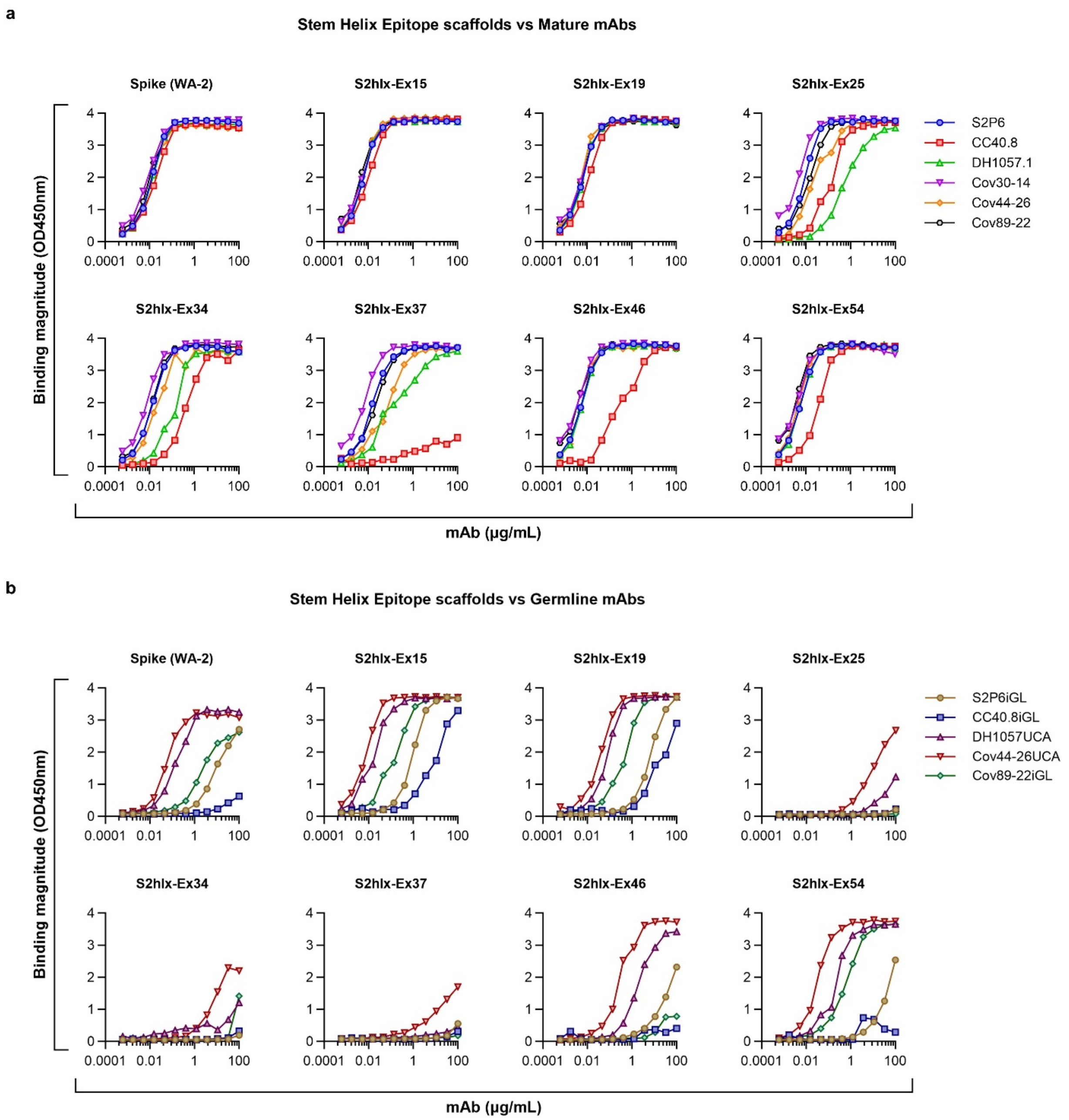

Supplementary Figure 17. Epitope scaffolds bind to diverse mature and germline antibodies that target the stem helix epitope. (a) ELISA binding of mAbs S2P6, DH1057.1, CC40.8, Cov30-14, Cov44-26, and Cov89-22 to S2hlx-Ex ESs and to Spike (WA-2) protein. **(b)** ELISA binding of mAbs S2P6iGL, DH1057UCA, CC40.8iGL, Cov44-26UCA, and Cov89-22iGL to S2hlx-Ex ESs and to Spike (WA-2) protein. Source data are provided as a Source Data file**.**

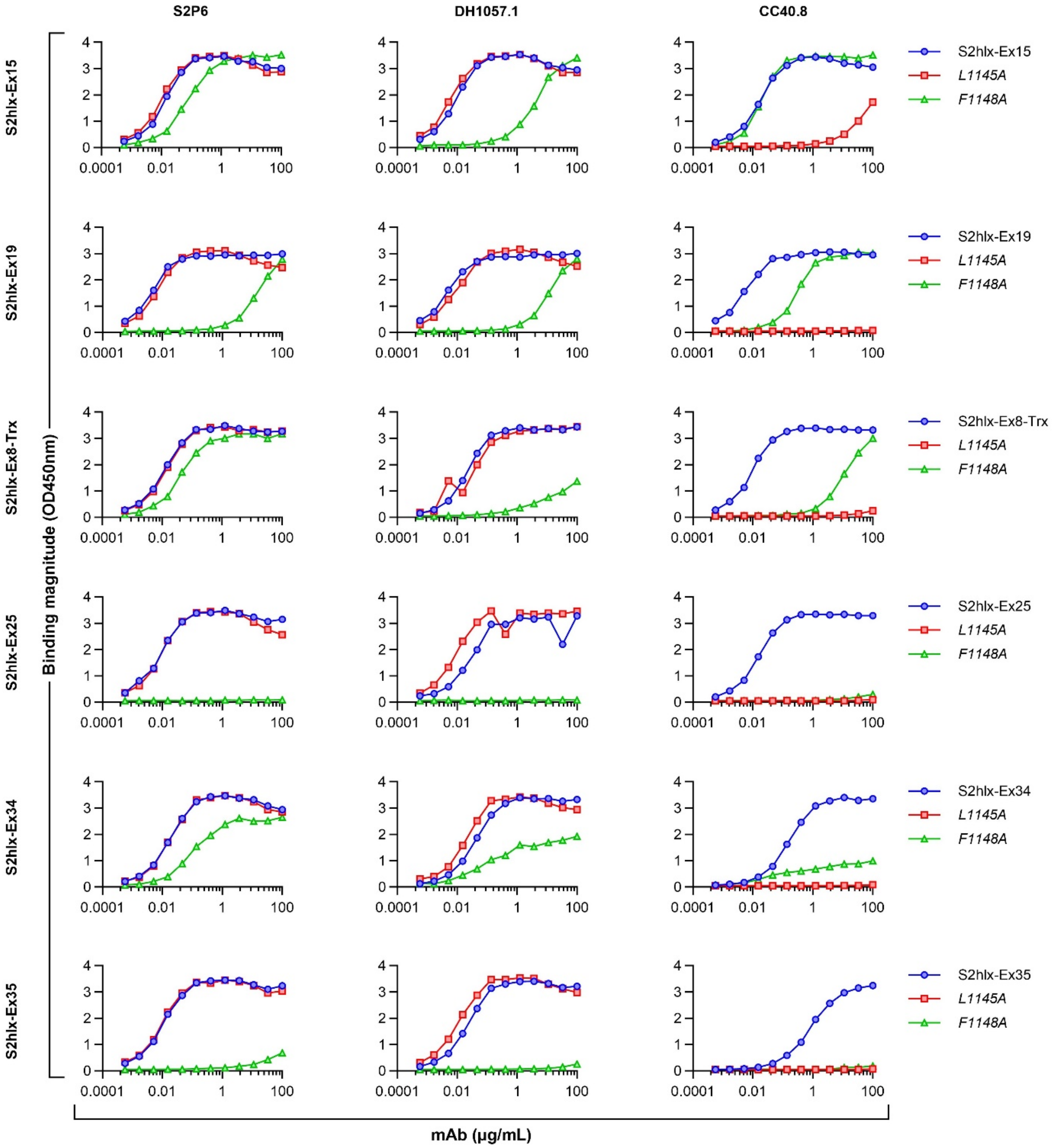

Supplementary Figure 18. Binding specificity of S2hlx-Ex epitope scaffolds for representative epitopes against the stem helix epitope. Indicated spike mutations known to affect the binding of stem helix were introduced in the respective epitope scaffolds. Source data are provided as a Source Data file.

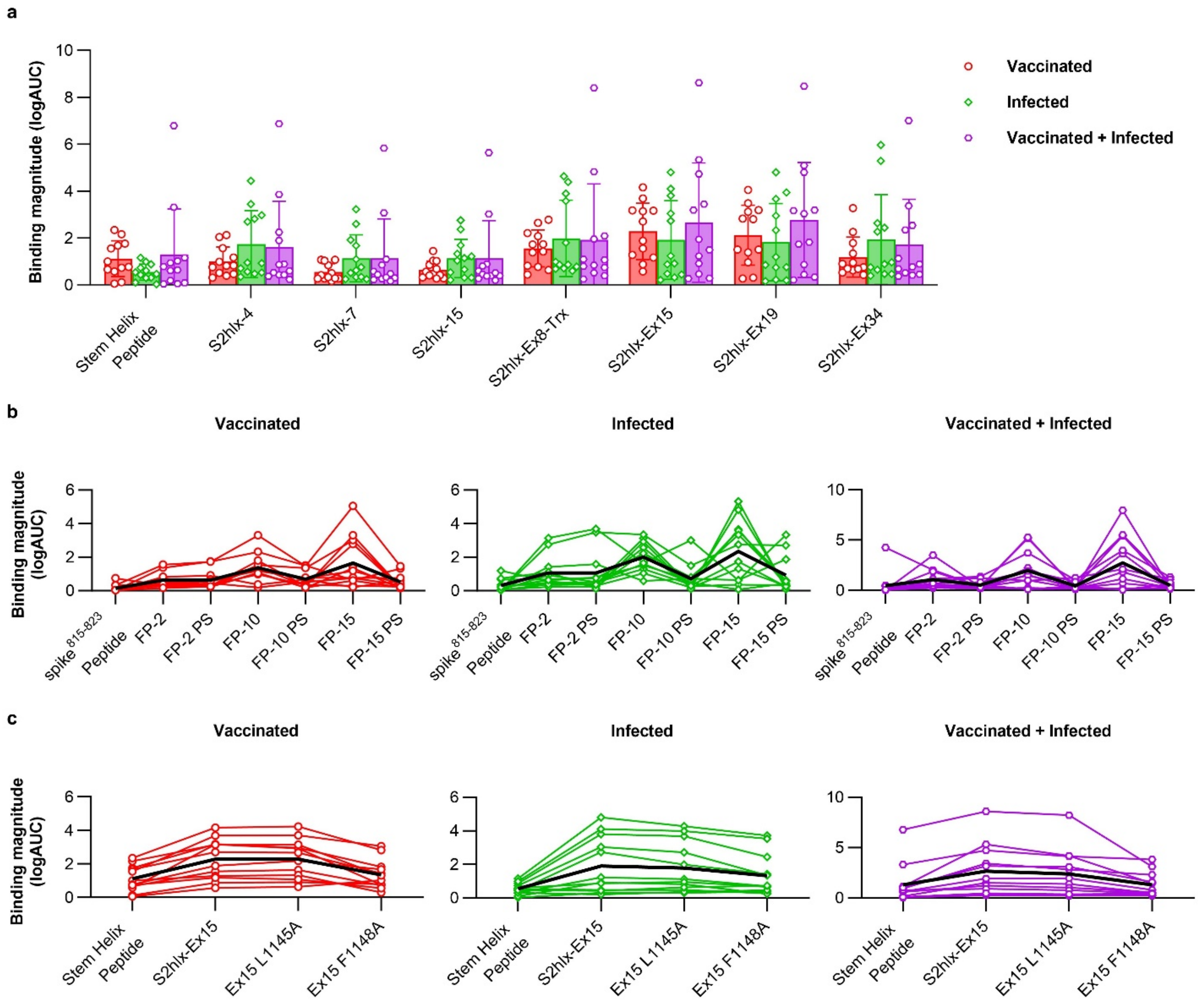

Supplementary Figure 19. Reactivity of designed epitope scaffolds to patient sera with pre-existing immunity to the SARS-CoV-2 spike. (a) Binding of stem helix peptide and select S2hlx and S2hlx-Ex epitope scaffolds. Bar plot values represents the mean +/- SD, with the individual samples (n=12 per group) shown in circles. **(b)** Binding of stem helix peptide, S2hlx-Ex15 and S2hlx-Ex15 epitope mutants that reduce binding to either the CC40.8-class of mAbs (LA) or to both the CC40.8 and S2P6 classes of mAbs (FA). **(c)** Binding to spike$^{815\text{-}823}$ epitope scaffolds and to the parent scaffolds they were derived from and that lack the grafted epitope. Lines represent individual measurements for each sample in the group. Source data are provided as a Source Data file**.**

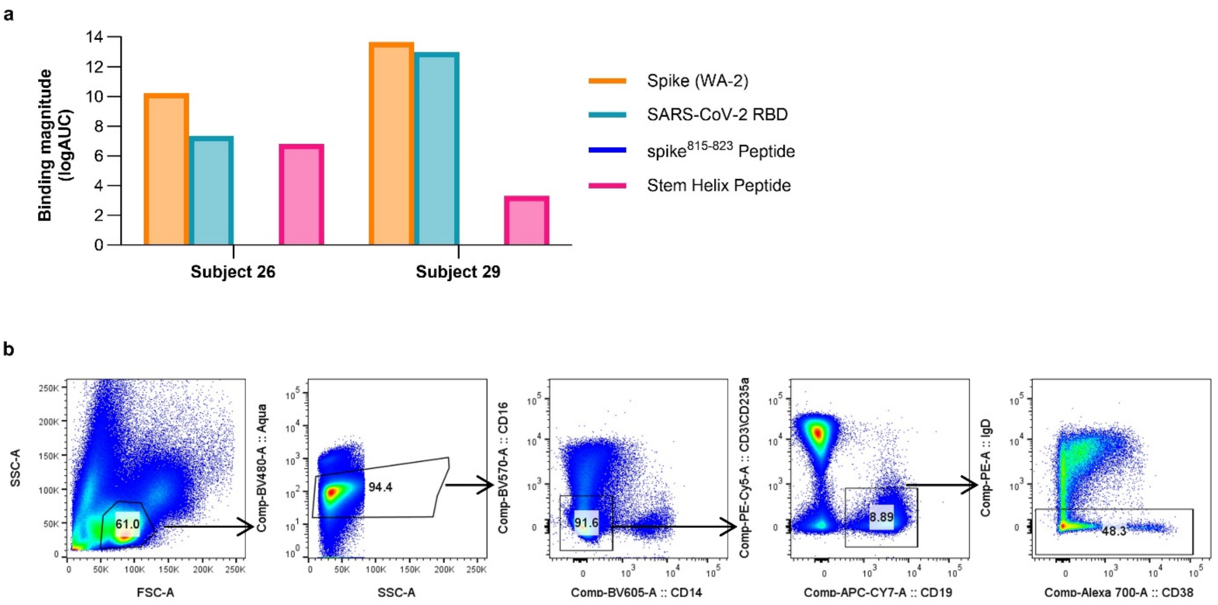

Supplementary Figure 20. Isolation of stem helix epitope-specific memory B cells from vaccinated then infected subjects with stem helix scaffolds. (a) ELISA binding of sera from indicated subjects with pre-existing SARS-CoV-2 immunity acquired by vaccination followed by infection to recombinant WA-2 spike and RBD or to synthetic peptides containing the spike$^{815-823}$ epitope (residues 808-833) or the stem helix (1140-1164). **(b)** Flow cytometric gating strategy for the isolation of memory B cells from PBMCs of subjects. Source data are provided as a Source Data file.

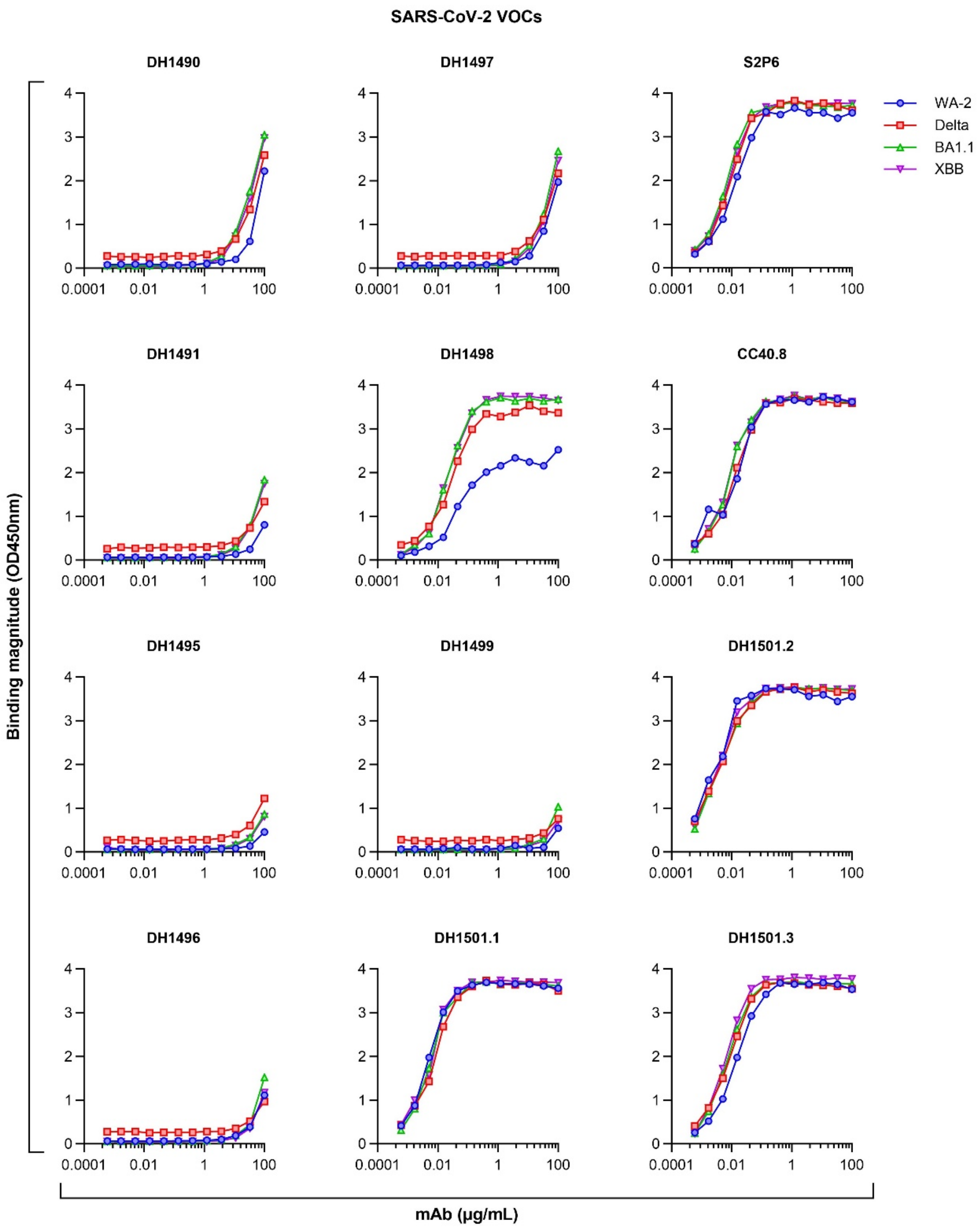

Supplementary Figure 21. ELISA binding of isolated stem helix antibodies to SARS-CoV-2 VOC spikes. Source data are provided as a Source Data file**.**

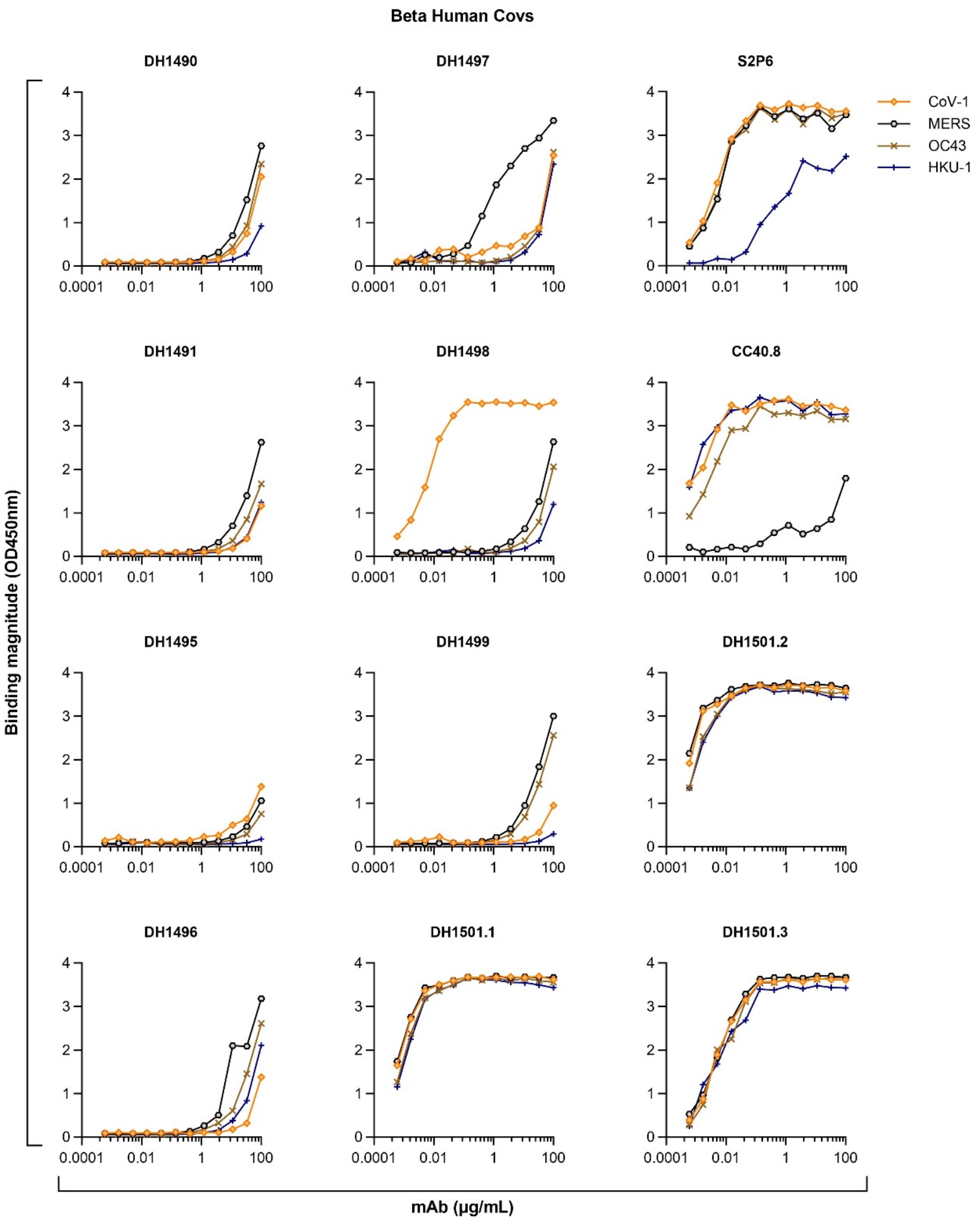

Supplementary Figure 22. ELISA binding of isolated stem helix antibodies to human betacoronavirus spikes. Source data are provided as a Source Data file**.**

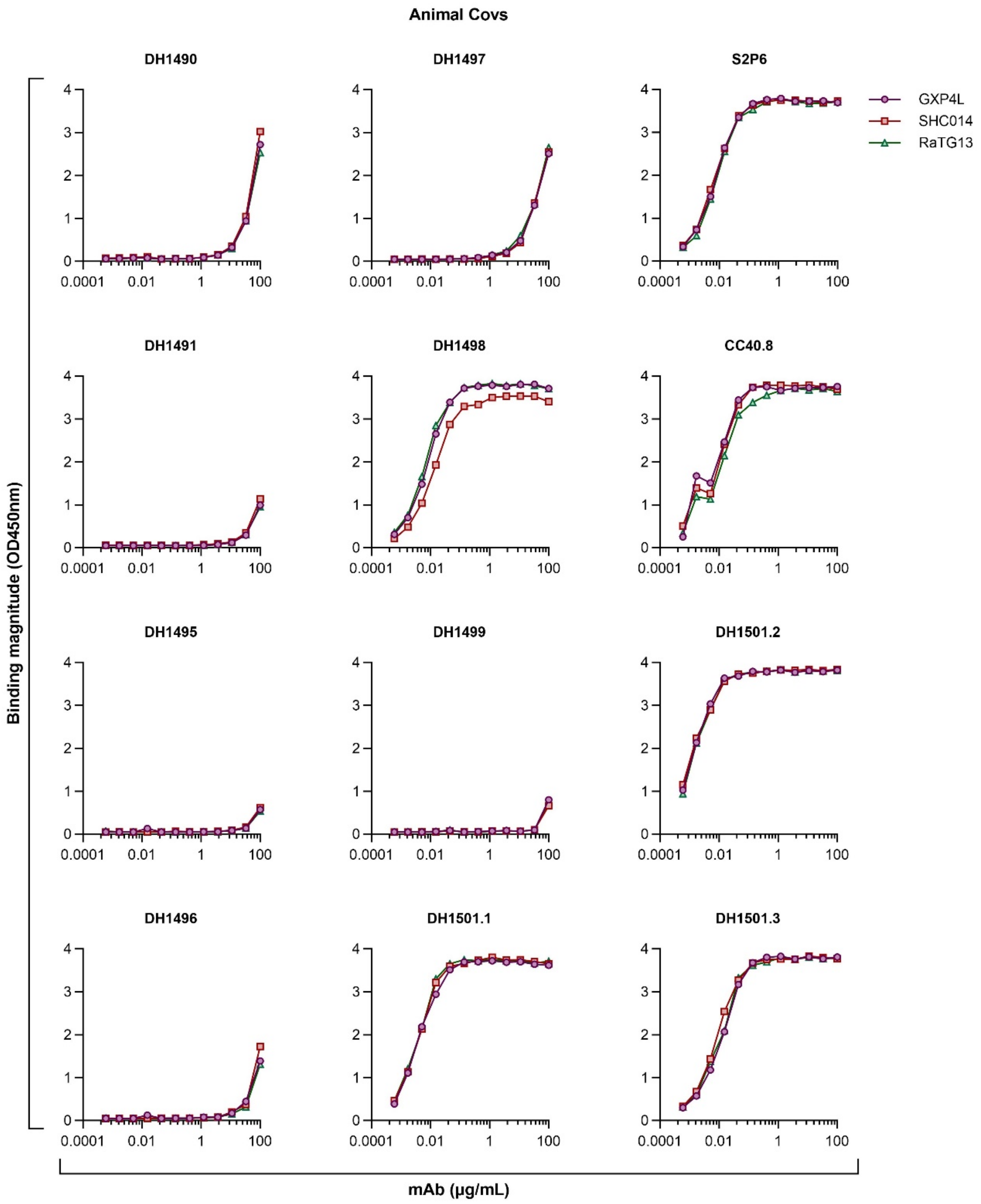

Supplementary Figure 23. ELISA binding of isolated stem helix antibodies to animal betacoronavirus spikes. Source data are provided as a Source Data file**.**

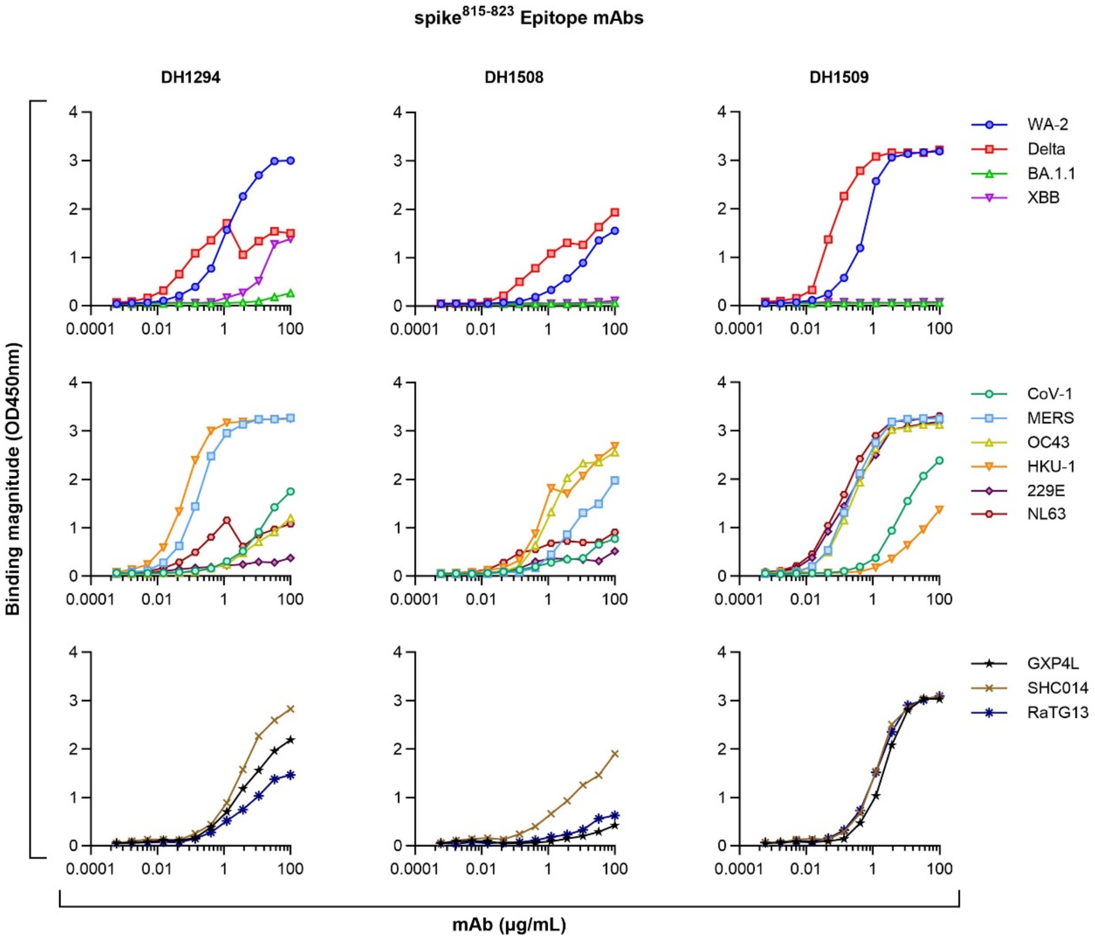

Supplementary Figure 24. ELISA binding of isolated spike$^{815\text{-}823}$ antibodies to human and animal coronavirus spikes. Source data are provided as a Source Data file**.**

a

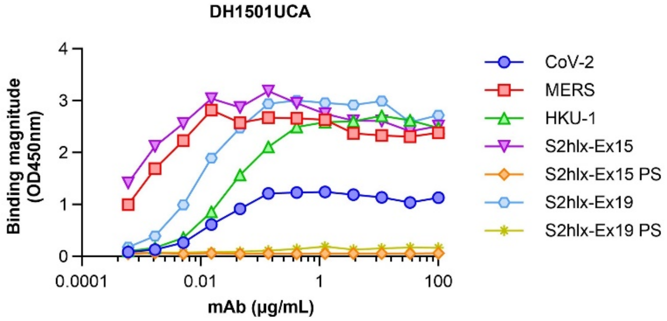

b

```
Heavy: IGHV1-46*01|IGHD1-26*01|IGHJ3*02

DH1501UCA QVQLVQSGAEVKKPGASVKVSCKASGYTFTSYYMHWVRQAPGQGLEWMGIINPSGGSTSYAQKFQGRVTMTRDTSTSTVYMELSSLRSEDTAVYYCARMSRYGAFDIWGQGTMVTVSS
DH1501.3
          ..................RL......D...NE.VQ.............L.....SG.AF.RN.....S.......R....D.T...Y.............A.T..L..........
DH1501.1
          E....E............R.......F...DH................L...T.VN.F...N.R...........K.D.L.VR..T.Q...M.......................L
DH1501.2
          E....E............RL......D..NNK.V...............L.....SG.AF........S.......R...LG.T..TY.............A.T....D.........

Light: IGKV3-20*01|IGKJ2*01

DH1501UCA EIVLTQSPGTLSLSPGERATLSCRASQSVSSSYLAWYQQKPGQAPRLLIYGASSRATGIPDRFSGSGSGTDFTLTISRLEPEDFAVYYCQQYGSSPPRYTFGQGTKLEIK
DH1501.3
          ........D.....L......F....ETID.R..........R.....MS.T.V......................T.............................AM.
DH1501.1
          .............................G.G.....................V..................S...N.V......M....N..................
DH1501.2
          ................D.........EN.GT..................S.T......V..........R.....................................DM.
```

Supplementary Figure 25. The unmutated common ancestor (UCA) of isolated stem helix antibodies DH1501.1, DH1501.2, and DH1501.3, binds to multiple human coronavirus spikes. (a) ELISA binding data. **(b)** Sequence alignment between the UCA and the mature antibodies. Source data are provided as a Source Data file**.**

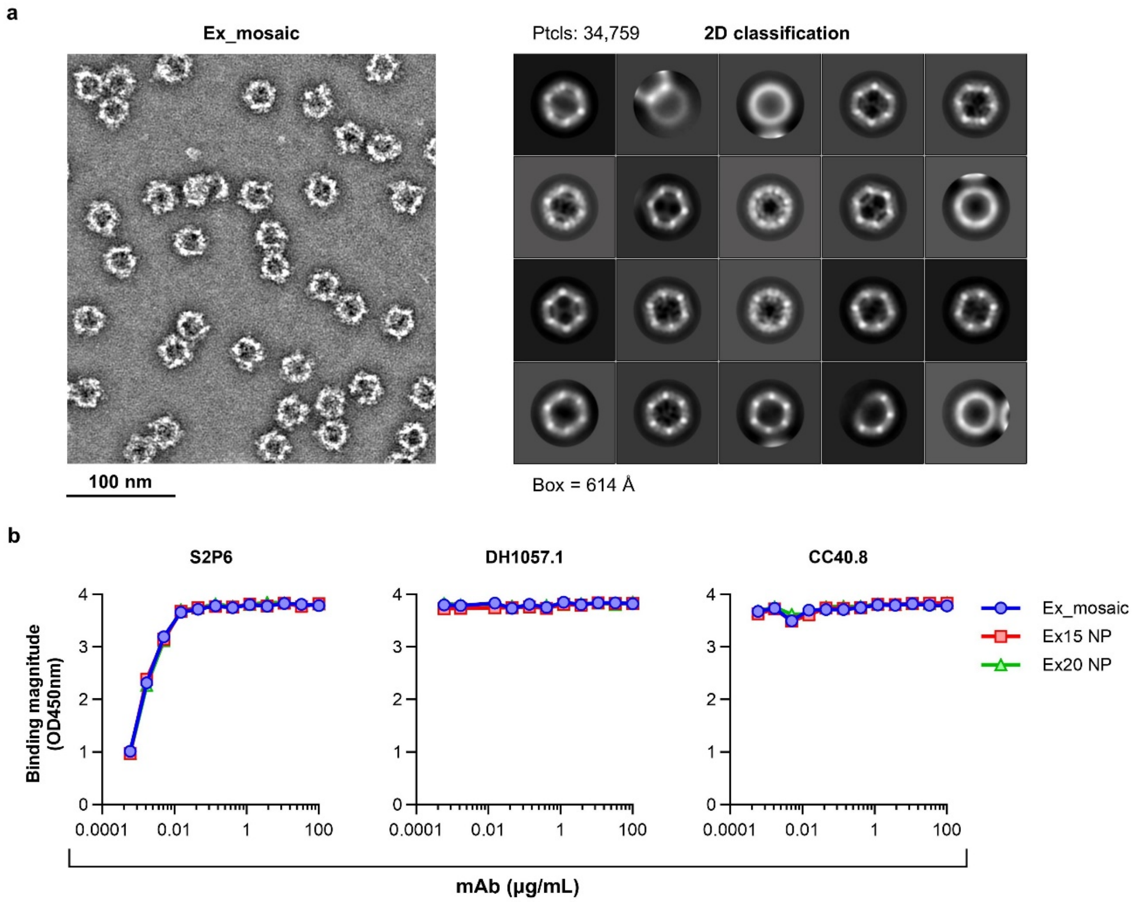

Supplementary Figure 26. Characterization of epitope scaffold nanoparticles. (a) NSEM sample image and 2D classification of the Ex_mosaic NP. **(b)** ELISA binding of mi03 nanoparticles displaying the indicated epitope scaffolds to stem helix antibodies S2P6, DH1057.1 and CC40.8. Source data are provided as a Source Data file**.**

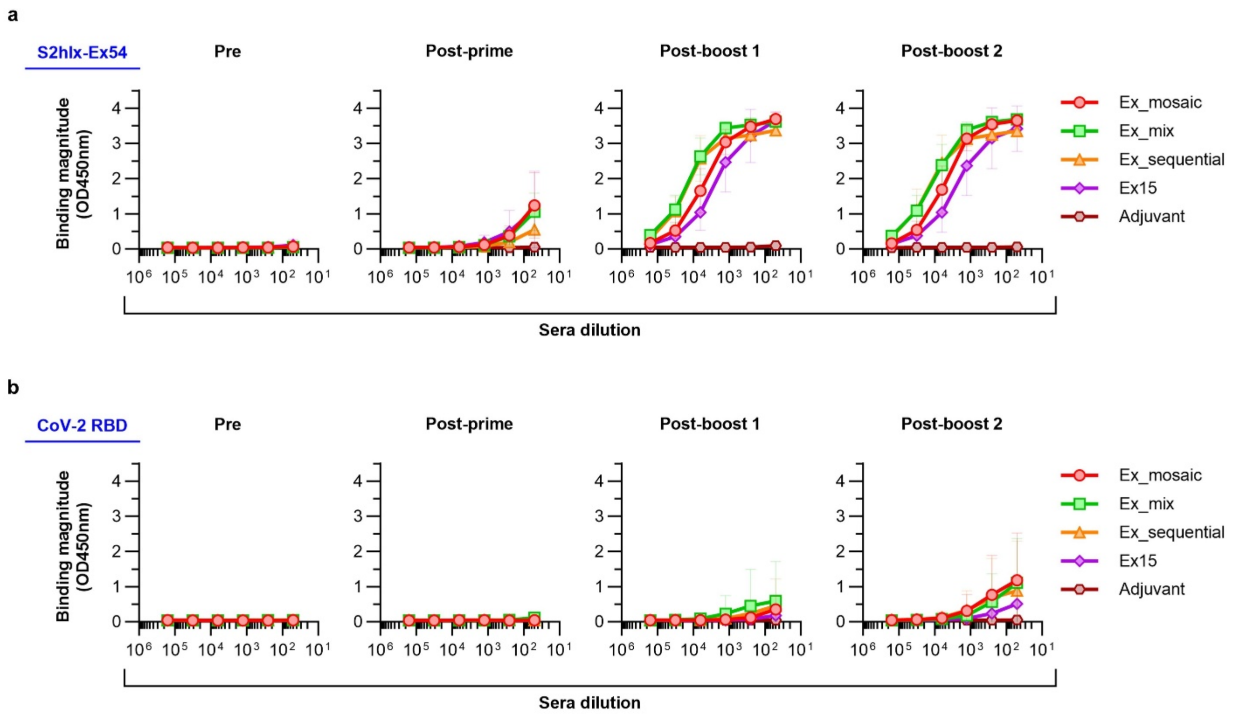

Supplementary Figure 27. Binding of sera from *BALBc* immunized mice with stem helix epitope scaffold nanoparticles to an epitope scaffold not used in the immunization and the RBD. (a) sera binding at indicated time points to a stem helix epitope scaffold S2hlx-Ex54. **(b)** sera binding at indicated time points to the RBD. Data are presented as the mean. Source data are provided as a Source Data file**.**

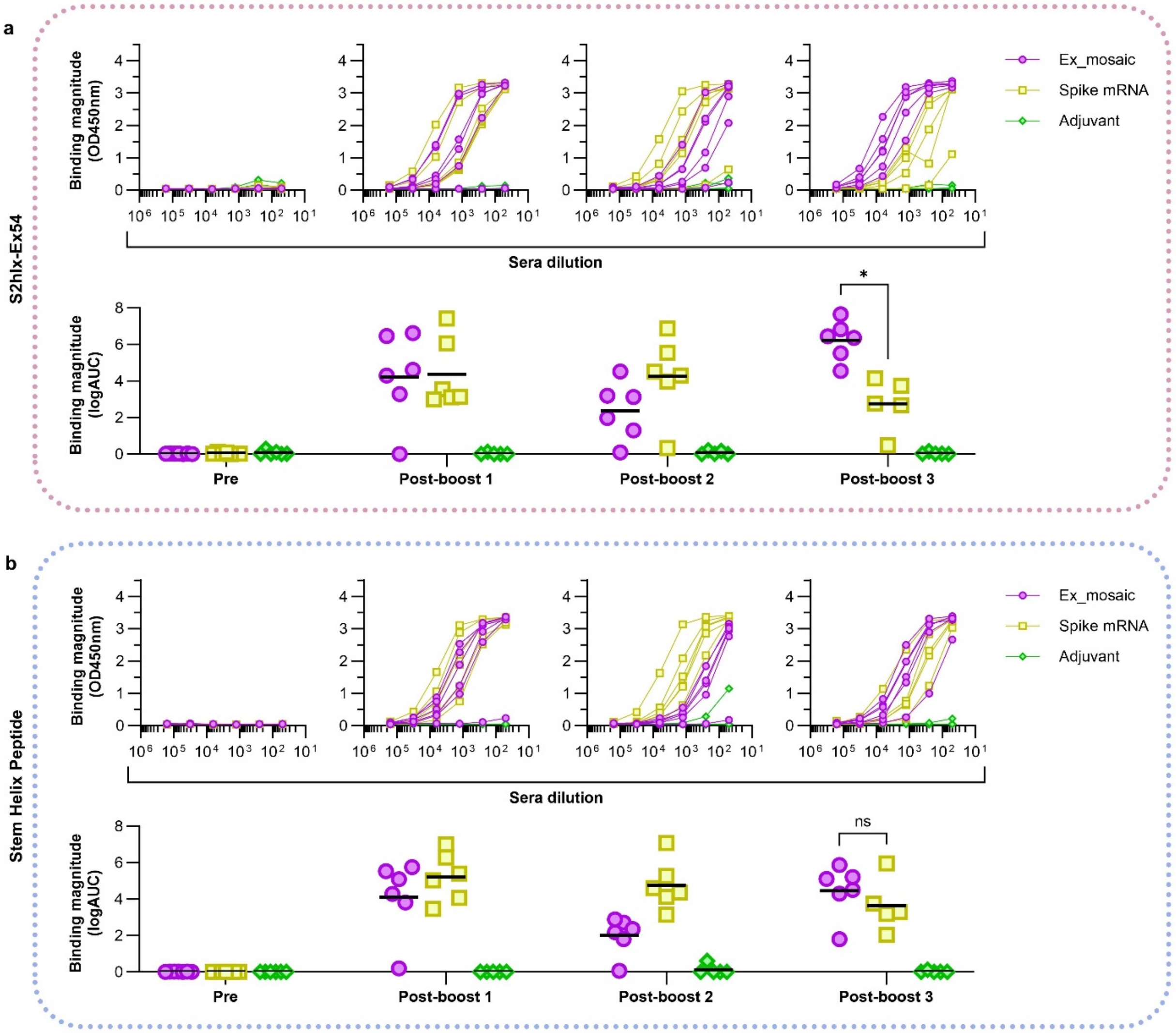

Supplementary Figure 28. Stem helix epitope specific binding of sera from *K18-ACE2* mice primed with spike mRNA and boosted with either the epitope scaffold mosaic NP or spike mRNA. (a) sera binding at indicated time points to a stem helix epitope scaffold not used in the immunizations (S2hlx-Ex54). **(b)** sera binding at indicated time points to a synthesized peptide encoding the stem helix epitope. Data are presented as the mean. Data points correspond to individual animals. F was calculated by two-way ANOVA using Geisser-Greenhouse correction and comparisons using Tukeys test. ns=not significant; * p=0.015. Source data are provided as a Source Data file.
