# Supplementary material for: Engineered immunogens to elicit antibodies against conserved coronavirus epitopes
Source: Nat Commun. 2023 Nov 30;14:7897. doi: 10.1038/s41467-023-43638-9 (PMC10689493; doi:10.1038/s41467-023-43638-9)
Supplement: Supplementary file 3 — Description of Additional Supplementary Files [file 41467_2023_43638_MOESM3_ESM.pdf]

Description of Additional Supplementary Files:

Supplementary Data 1: Design characteristics and initial experimental screening data for all the epitope scaffolds tested experimentally. FP, S2hlx and S2hlx-Ex epitope scaffolds originate from diverse parent proteins. Epitope scaffolds were expressed recombinantly in E. Coli and tested for binding to spike815-823 and stem helix targeting mAbs by ELISA (+++ denotes high affinity; ++ moderate affinity; + low affinity). SC= side chain; BB=backbone; AF= AlphaFold2.
