# Supplementary material for: Engineered immunogens to elicit antibodies against conserved coronavirus epitopes
Source: Nat Commun. 2023 Nov 30;14:7897. doi: 10.1038/s41467-023-43638-9 (PMC10689493; doi:10.1038/s41467-023-43638-9)
Supplement: Supplementary file 5 — Reporting Summary [file 41467_2023_43638_MOESM5_ESM.pdf]

nature portfolio

Corresponding author(s): Azoitei ML

Last updated by author(s): Oct 17, 2023

Reporting Summary

Nature Portfolio wishes to improve the reproducibility of the work that we publish. This form provides structure for consistency and transparency in reporting. For further information on Nature Portfolio policies, see our Editorial Policies and the Editorial Policy Checklist.

Statistics

For all statistical analyses, confirm that the following items are present in the figure legend, table legend, main text, or Methods section.

n/a	Confirmed	
☐	☒	The exact sample size (*n*) for each experimental group/condition, given as a discrete number and unit of measurement
☐	☒	A statement on whether measurements were taken from distinct samples or whether the same sample was measured repeatedly
☐	☒	The statistical test(s) used AND whether they are one- or two-sided<br>*Only common tests should be described solely by name; describe more complex techniques in the Methods section.*
☒	☐	A description of all covariates tested
☒	☐	A description of any assumptions or corrections, such as tests of normality and adjustment for multiple comparisons
☐	☒	A full description of the statistical parameters including central tendency (e.g. means) or other basic estimates (e.g. regression coefficient) AND variation (e.g. standard deviation) or associated estimates of uncertainty (e.g. confidence intervals)
☐	☒	For null hypothesis testing, the test statistic (e.g. *F*, *t*, *r*) with confidence intervals, effect sizes, degrees of freedom and *P* value noted<br>*Give P values as exact values whenever suitable.*
☒	☐	For Bayesian analysis, information on the choice of priors and Markov chain Monte Carlo settings
☒	☐	For hierarchical and complex designs, identification of the appropriate level for tests and full reporting of outcomes
☒	☐	Estimates of effect sizes (e.g. Cohen's *d*, Pearson's *r*), indicating how they were calculated

Our web collection on statistics for biologists contains articles on many of the points above.

Software and code

Policy information about availability of computer code

Data collection	Rosetta v3.9; ProteinMPNN; AlphaFold v2.1
Data analysis	Prism version 10.0.0 (Graphpad); PyMol v2.5.5 (Schroedinger); Excel 16.77.1 (Microsoft); FlowJo v10 (BD)

For manuscripts utilizing custom algorithms or software that are central to the research but not yet described in published literature, software must be made available to editors and reviewers. We strongly encourage code deposition in a community repository (e.g. GitHub). See the Nature Portfolio guidelines for submitting code & software for further information.

Data

Policy information about availability of data

All manuscripts must include a data availability statement. This statement should provide the following information, where applicable:

- Accession codes, unique identifiers, or web links for publicly available datasets
- A description of any restrictions on data availability
- For clinical datasets or third party data, please ensure that the statement adheres to our policy

Crystal structures have been deposited in the PDB under accession numbers 8F5I (https://www.rcsb.org/structure/8F5I), 8FDO (https://www.rcsb.org/structure/8FDO) and 8F5H (https://www.rcsb.org/structure/8F5H). Plasmids encoding the engineered epitope scaffolds can be obtained through an MTA from the corresponding author. Source data are provided with this paper.

Research involving human participants, their data, or biological material

Policy information about studies with human participants or human data. See also policy information about sex, gender (identity/presentation), and sexual orientation and race, ethnicity and racism.

Reporting on sex and gender	Sex and gender were not considered in this paper. Researchers were blinded to this information.
Reporting on race, ethnicity, or other socially relevant groupings	None of these variables were used in the analysis. Researchers were blinded to this information.
Population characteristics	No covariate data is reported in this paper. Researchers were blinded to this information.
Recruitment	Human sera samples were selected from study participants previously enrolled in the Molecular and Epidemiological Study of Suspected Infection protocol (MESSI, IRB Pro00100241) at Duke University. Participants were recruited from the Durham, NC community through various avenues, including Duke Hospital inpatients and outpatients, Duke employees and housing shelters. Samples were selected for analysis in this study from participants who had seroconverted and had symptom onset more than 10 days prior to sample collection.
Ethics oversight	Study protocols were approved by the Duke University Health System Institutional Review Board (IRB)

Note that full information on the approval of the study protocol must also be provided in the manuscript.

Field-specific reporting

Please select the one below that is the best fit for your research. If you are not sure, read the appropriate sections before making your selection.

☒ Life sciences ☐ Behavioural & social sciences ☐ Ecological, evolutionary & environmental sciences

For a reference copy of the document with all sections, see nature.com/documents/nr-reporting-summary-flat.pdf

Life sciences study design

All studies must disclose on these points even when the disclosure is negative.

Sample size	No sample size calculation was performed. Samples were limited by availability.
Data exclusions	No data was excluded
Replication	All in vitro and cellular experiments were repeated at least twice independently. Mouse immunization studies were only conducted once due to resource availability.
Randomization	All animals were female and matched by age so no randomization was necessary.
Blinding	Investigators were not blinded to data collection and analysis. Blinding is not typically performed for animal studies that are analyzed the same way.

Reporting for specific materials, systems and methods

We require information from authors about some types of materials, experimental systems and methods used in many studies. Here, indicate whether each material, system or method listed is relevant to your study. If you are not sure if a list item applies to your research, read the appropriate section before selecting a response.

Materials & experimental systems

n/a	Involved in the study	
☐	☒	Antibodies
☐	☒	Eukaryotic cell lines
☒	☐	Palaeontology and archaeology
☐	☒	Animals and other organisms
☒	☐	Clinical data
☒	☐	Dual use research of concern
☒	☐	Plants

Methods

n/a	Involved in the study	
☒	☐	ChIP-seq
☐	☒	Flow cytometry
☒	☐	MRI-based neuroimaging

Antibodies

Antibodies used	PE anti-human IgD (clone IA6-2, BD Biosciences), PE-TXRD anti-human CD10 (clone HI10A, BD Biosciences), PE-Cy5 anti-human CD3

	(clone HIT3a, BD Biosciences), PE-Cy7 anti-human C27 (clone O323, ThermoFisher Scientific), AlexaFluor 700 anti-human CD38 (clone LS198-4-3, Beckman Coulter), APC-Cy7 anti-human CD19 (clone SJ25C1, BD Biosciences), BV570 anti-human CD16 (clone 3G8, Biolegend), BV605 anti-human CD14 (clone M5E2, Biolegend), and BV711 anti-human IgM (clone G20-127, BD Biosciences). goat anti mouse IgG-HRP, 1:10,000 dilution, Jackson ImmunoResearch Laboratories; Code Number: 115-035-071; Lot number: 158206; or goat anti human IgG-HRP, 1:15,000 dilution, Jackson ImmunoResearch Laboratories, Code Number: 109-035-098; Lot number: 154823. anti-CD107a-FITC (BD Biosciences, clone H4A3). anti-CD56-PECy7 (BD Biosciences, clone NCAM16.2). anti-CD16-PacBlue (BD Biosciences, clone 3G8). anti-CD69-BV785 (Biolegend, Clone FN50)
Validation	Antibodies are validated by manufacturers and positive and negative controls are described in the text.

Eukaryotic cell lines

Policy information about cell lines and Sex and Gender in Research

Cell line source(s)	293T (ATCC); Vero E6 (ATCC); 293T/ACE2-MF cells (from Drs. Mike Farzan and Huihui Mu at The Scripps Research Institute)
Authentication	None of the cells were authenticated
Mycoplasma contamination	Cells tested negative for microplasma contamination
Commonly misidentified lines (See ICLAC register)	not used

Animals and other research organisms

Policy information about studies involving animals; ARRIVE guidelines recommended for reporting animal research, and Sex and Gender in Research

Laboratory animals	Balb/c mice (2-3 months old); K18-ACE2 mice (14 months old)
Wild animals	No wild animals were used.
Reporting on sex	All mice used were femlaes.
Field-collected samples	No field-collected samples were used.
Ethics oversight	Mouse studies were performed according to the recommendations for the care and use of animals by the Office of Laboratory Animal Welfare (OLAW), National Institutes of Health, and the Institutional Animal Care and Use Committee (IACUC) at the University of North Carolina (UNC permit no. A-3410-01).

Note that full information on the approval of the study protocol must also be provided in the manuscript.

Plants

Seed stocks	No plants were used in this study.
Novel plant genotypes	n/a
Authentication	n/a

Flow Cytometry

Plots

Confirm that:

- [x] The axis labels state the marker and fluorochrome used (e.g. CD4-FITC).
- [x] The axis scales are clearly visible. Include numbers along axes only for bottom left plot of group (a 'group' is an analysis of identical markers).
- [x] All plots are contour plots with outliers or pseudocolor plots.
- [x] A numerical value for number of cells or percentage (with statistics) is provided.

Methodology

Sample preparation	Cryopreserved PBMCs were thawed in warm RPMI-1640 containing 10% FBS, then counted
Instrument	BD S6 cell sorter (BD Biosciences).
Software	FlowJo v10.8 (BD Biosciences).
Cell population abundance	This is described in the methods
Gating strategy	This is described in the methods

☒ Tick this box to confirm that a figure exemplifying the gating strategy is provided in the Supplementary Information.
